# Raising “Antiracist Disruptors”: Illuminating Socialization Practices that Support Antiracism in Multiracial Households

**DOI:** 10.1007/s12552-023-09389-4

**Published:** 2023-02-01

**Authors:** Victoria A. Vezaldenos, Laura-Ann Jacobs, Deborah Rivas-Drake

**Affiliations:** University of Michigan, Ann Arbor, USA

**Keywords:** Multiracial families, Antiracist socialization, Ethnic-racial socialization, Parenting multiracial children, Consensual qualitative analysis

## Abstract

Although an emerging body of literature has advanced our knowledge of how monoracial parents can support their multiracial children in understanding the ethnic-racial identities they hold, there is a dearth of research exploring how parents socialize their children towards antiracism. Drawing from ten interviews with monoracial parents of multiracial children, this paper illuminates how parents leverage multiracial socialization practices, as identified in previous academic research, to instill an antiracist orientation in their children. Using consensual qualitative analyses, we find that although all parents had a vested interest in the wellbeing and identity development of their multiracial children, parents qualitatively differed in their ability and willingness to instill an antiracist orientation in their children. Specifically, parents in our sample exhibited five approaches to multiracial socialization, ranging from those that reinforced dominant racial ideologies to those that explicitly aimed to prepare youth to become antiracist activists. We also describe how monoracial parents’ lived experiences are implicated in their engagement in multiracial socialization practices, especially those that better position them to prepare their children to engage in antiracism. Our findings illuminate how monoracial parents may engage in a repertoire of strategies in order to foster antiracism in multiracial children, molding the next generation of “antiracist disruptors.”

## Introduction

In the last decade, the population of multiracial youth has increased by 276% and is projected to further increase in the decade to come (U.S. Census Bureau, 2021).^1^ Multiracial youths’ racial knowledge has important implications for their life outcomes, as their understanding of race and racial identity can aid in better outcomes for their mental, physical, and emotional wellbeing, psychological adjustment, and political participation (e.g., Christophe, 2021; Fisher et al., 2014; Goodhines et al., 2020; Jackson et al., 2012; Rockquemore & Brunsma, 2007; Tabb, 2016). Recent studies have examined how parents of multiracial children can support their youths’ understanding of their own ethnic-racial identities through various types of socialization practices (e.g., Atkin & Jackson, 2021; Atkin & Yoo, 2019; Atkin et al., 2022; Csizmadia & Atkin, 2022; LaBarrie, 2017; Lorenzo-Blanco et al., 2013). Because monoracial parents do not share the same ethnic-racial identity as their multiracial children, supporting their children to explore and accommodate various ethnic-racial identities can be challenging.

Although an emerging body of literature has advanced our knowledge of how monoracial parents can support their multiracial children in understanding the ethnic-racial identities they hold (e.g., Atkin & Jackson, 2021; Atkin & Yoo, 2019; Csizmadia & Atkin, 2022), there is a dearth of research exploring the practices parents use to address racism and antiracism within multiracial homes. Parents' socialization practices may not only inform multiracial youths’ self-understanding in terms of their identities but also serve to resist or reinforce dominant racial ideologies. For example, how do multiracial families discuss racialized issues pertaining to groups not represented in their home? How do they broach ideas of systemic vis-à-vis interpersonal racism? The extent to which multiracial youth are exposed to such ideas and issues may have implications not only for promoting or inhibiting their identity development but also their orientation toward antiracism (e.g., acknowledging racism as historical and systemic and seeking to dismantle these structures to promote equal opportunity for ethnic-racial groups; Berman & Paradies, 2010) and capacity for antiracism actions (e.g., taking action to dismantle racism interpersonally, communally, and/or politically; Bañales et al., 2019).

The current study contributes insights into monoracial parents’ multiracial socialization practices. In particular, we sought to explore how monoracial parents may promote the development of an antiracist orientation and capacity for antiracism action in their multiracial children. We identified multiracial socialization practices that can serve to both perpetuate and resist dominant ideologies and describe how parents’ lived experiences inform such practices.

### Extant Multiracial Socialization Practices

According to Csizmadia and Atkin (2022; see also Atkin & Yoo, 2019), there are two qualitatively distinct categories of multiracial socialization practices: *racial-ethnic socialization* (RES) and *family support of multiracial experiences* (FSME). Multiracial RES involves several practices that teach children about different ethnic-racial group memberships and experiences, as articulated byCsizmadia and Atkin (2022).^2^ For example, *cultural socialization*, as it pertains to multiracial households, involves parents engaging youth in conversation or exploration of one or more of their multiple cultural heritages, often with the goal of instilling racial pride. Parents also engage in *preparation for bia*s socialization with their multiracial children for both monoracial and multiracial forms of bias they may experience from both in-groups and out-groups.

*Multiracial identity socialization* involves parental messages that emphasize multiraciality as a positive lived experience. More subtly, *exposure to diversity* entails having their children engage with diverse ethnic-racial groups through their community context, extracurricular engagements, or forms of media without explicitly addressing racial difference. Sometimes, parents may engage in *negative socialization*, instilling prejudicial attitudes toward particular ethnic-racial groups, sometimes groups of which their children are themselves a member. These messages may lead to the perpetuation and internalization of negative stereotypes, which can adversely impact youth adjustment (Ayón, 2016; Hughes et al., 2006).

Parents may engage their children in *race-conscious socialization*, teaching them to treat people from other ethnic-racial groups equally while also recognizing racism as systemic and pervasive (see also Atkin et al., 2022). *Diversity appreciation* involves parents teaching their children to appreciate cultural diversity and ethnic-racial differences without explicitly naming or discussing racism and discrimination (see also Atkin et al., 2022). Parents instill ideologies in their children by explaining that race does not matter and people should not see skin color via *colorblind (or color-evasive) socialization.* Parents that do not address race or racism with their children are engaging in *silent socialization*. Parents who teach their children that people can overcome discrimination and achieve racial equality through hard work are engaging in *egalitarian socialization* (see also Hughes et al., 2006). These messages convey to children that race should not matter in society and that to overcome racism people need to assimilate to the cultural mainstream and strive for meritocratic excellence (Aldana & Byrd, 2015; Hughes et al., 2006; Huguley et al., 2019). Finally, parents may tell their children to be wary of certain ethnic-racial groups via *promotion of mistrust* socialization. These messages have been associated with poor psychological adjustment as evidenced by greater prevalence of depressive symptoms (Atkin et al., 2019; Liu & Lau, 2013; Park et al., 2020).

Distinct from multiracial RES, Family Support of Multiracial Experiences (FSME) practices are more tailored to validating and supporting their identity-related experiences of multiracial children (Atkin & Jackson, 2021; Atkin et al., 2022; Csizmadia & Atkin, 2022); these practices are by definition unique to multiracial families. More specifically, parents that engaged in *multiracial identity expression support* were supportive of their children exploring some or all their ethnic-racial heritages and choosing how they wanted to identify. Parents that acknowledge multiraciality as a unique lived experience and positionality are engaging in multiracial *conscious support*. *Multiple heritage validation* occurs when parents are supportive of their child’s belonging to the multiple monoracial groups with which they share an identity. When children express discomfort or distress about not feeling close to their parents or their culture, parents express *connection support* by validating these feelings. Finally, parents that acknowledge and validate their multiracial children’s experiences with discrimination and racism engage in *racial discrimination support*.

Importantly, Csizmadia and Atkin (2022) explain that RES and FSME socialization practices differ such that RES practices impart messages to youth about race, ethnicity, or culture, whereas FSME practices are more about parents validating and supporting the unique lived experiences of their multiracial children. This extensive list of socialization practices yields important insights into parental influences on the development of multiracial children’s ethnic-racial identities. However, it is as yet unclear how these practices might map onto the perpetuation of racism or the promotion of antiracism (i.e., disruption of racism). For example, it stands to reason that race-conscious socialization (RES), preparation for bias (RES), and racial discrimination support (one type of FSME) may all be part of a process of teaching multiracial children to understand racism; however, the extent to which it would perpetuate or disrupt racism lies in the nature of the exchanges themselves, including the attributions made about the causes of racism. Color-evasive socialization practices, on the other hand, serve to perpetuate the idea that race should not be noticed, leaving youth underprepared to recognize and then challenge or disrupt racism when they encounter it. Consequently, this kind of socialization would be less effective in preparing multiracial youth to hold antiracist dispositions and engage in antiracist behaviors.

### Multiracial Socialization and Preparation for Antiracism

In the current study, we sought to answer the following research questions: *How do monoracial parents of multiracial children socialize their youth in ways that either perpetuate or disrupt racism? What lived experiences may inform these practices?* We sought to build on Csizmadia and Atkin’s (2022) framework for RES and FSME in multiracial households by examining how monoracial parents may perpetuate racist ideologies in the home, and conversely, what strategies they might use to foster an antiracist orientation in their multiracial children (i.e., be prepared to recognize and challenge racism). There are various possibilities for how monoracial parents may socialize their multiracial children about racism. One is that as parents encourage their children to examine and explore the multiple heritages to which they belong, they may simultaneously send negative messages that reinforce dominant ideologies about race and place the blame for unequal opportunities on individuals. Another is the case where monoracial parents may be making a concerted effort to engage their child in broader conversations regarding racial justice for a variety of ethnic-racial groups and encouraging their youth to recognize and resist systemic racism, as has been theorized in antiracist socialization among monoracial families (e.g., Bañales & Rivas-Drake, 2022; Hazelbaker et al., 2022; H. Lee et al., 2022). The latter possibility has been underexamined in extant literature centering the experiences of multiracial families. This may be perhaps due in part to an assumption that multiracial households are more aware or knowledgeable of racism and antiracism. As interracial unions became more common and the population of multiracial individuals proliferated, many believed (and continue to believe) that the U.S. was entering a “post-racial” society as historical racial categories were being challenged (Lee & Bean, 2012). Indeed, the mere existence of multiracial families itself challenges racist ideologies that have historically manifested through anti-miscegenation laws and hypodescent (Ho et al., 2011; Lee & Bean, 2012; Rockquemore & Brunsma, 2002, 2007; Root, 1990, 1996). However, being in an interracial relationship or heading a multiracial household does not necessarily disrupt the socialization of racist ideas and orientations (Brooks & Morrison, 2022; Gruenes, 2018; Pryor, 2018), as there are important individual differences in parents’ own understandings of the legacy of these racist laws and policies as well as their understanding of racism as a systemic issue, more generally.

Parents' own lived experiences position them in particular ways that shape how they address the needs of their multiracial children when broaching topics of race and ethnic-racial identity within the home (Atkin & Yoo, 2019; Lorenzo-Blanco et al., 2013; Miller, 2020; O’donoghue, 2005; Rondilla et al., 2017; Rockquemore & Brunsma, 2007; Talbot, 2008). For example, monoracial white parents sometimes struggle to prepare their multiracial children for racial bias as they themselves have never navigated the world as a person of color (O’donoghue, 2005). However, monoracial black parents may draw from their direct knowledge of racism and discrimination and share these perspectives with their multiracial children to prepare them for racial bias (Csizmadia & Atkin, 2022). Thus, parents’ lived experiences may inform their approach to addressing race and racism with their multiracial children.

In addition to Csizmadia and Atkin’s (2022) aforementioned work on multiracial socialization, we further ground our study in the tenets of critical multiracial theory (MultiCrit; Harris, 2016) and Renn’s (2003) and Miville’s (2005) application of the Bronfenbrenner’s (1995) ecological systems model of child development for multiracial youth. MultiCrit disrupts dominant monoracial paradigms and centers the experiences of multiracial students to reveal racist structures and enact social justice (Harris, 2016). We rely on Harris’s (2016) MultiCrit framework primarily to explicitly center the nuanced lived experiences of multiracial youth by challenging a monoracial paradigm to race and attending to racism, monoracism, and colorism in our analyses of the data. The MultiCrit tenets (Harris, 2016) also allow us to reflect on how multiracial children’s racialized experiences may align with and/or differ from those of their monoracial parents.

Further, we draw on Miville (2005) and Renn (2003) by noting the importance of critical places, people, and periods for the development of multiracial people in the current analyses. We also attend to the chronosystem from the ecological systems model of development, which highlights the critical role of the current sociopolitical and historical context (Bronfenbrenner, 1995). In 2020, the U.S. came to grips not only with the COVID-19 pandemic, but also the pandemic of systemic, anti-Black racism following the murder of George Floyd (Ho, 2021; Laurencin & Walker, 2020). As protests and activist movements erupted across the country and news feeds were filled with racialized reports of the incidents, conversations about race and racism began to trickle into homes, workplaces, and schools. Simultaneously, race-related issues were being further inflamed by Trump-era rhetoric resulting in an uptick in anti-Asian violence, outrage at the separation of children and parents at the U.S./Mexico border, and a siege of the U.S. capitol by white supremacists (Ho, 2021; Johnson, 2022; Katner, 2021). Indeed, many parents in interviews for the larger project from which this study is based brought up the Black Lives Matter movement, children in cages, anti-Asian violence, the murders of Ahmaud Arbery and Breonna Taylor, the Kyle Rittenhouse trial, police brutality, the January 6th insurrection, and more, indicating the salience of the current sociopolitical context.

## Method

### Participants

We draw on qualitative interviews with monoracial parents of multiracial children conducted as a part of the Standing uP Against Racism and Xenophobia (SPARX) Project. SPARX is a community-oriented, action research project that aims to develop resources for parents and educators to assist them in addressing racism and xenophobia with the youth in their lives. The larger project includes a national sample of sixty-two parents recruited from rural, urban, and suburban U.S. communities via Qualtrics between November 2021 and February 2022. Prior to interviewing, participants were asked if they were “comfortable speaking on the topics of race, racism, immigration, and xenophobia” to ensure that they would be responsive to the interview protocol. Twelve parents in our sample identified as residing in multiracial households (i.e., two or more ethnic-racial identities were represented). In this study, we further refined our sample to include only monoracial parents of multiracial children, because as noted, households involving monoracial partners in an interracial relationship provide a unique context to assess socialization of multiracial youth (Christophe et al., 2021; Miller, 2020; O’donoghue, 2005). Participants in the present sample were interviewed between December 2021 and February 2022.

All participants were mothers with biracial children. Participants are parents of multiracial Asian/Latino (n = 2), Asian/white (n = 2), Black/white (n = 2), Black/Latino (n = 1), Native American/white (n = 1), Native American/Black (n = 1), and Native American/Latino (n = 1) households. The mothers identified as monoracial white (n = 2), Black (n = 1), Latino (n = 1), Asian (n = 4), and Native American (n = 2). Three mothers were parents to children with different fathers, resulting in two of the households having children with ethnic-racial identities that were different from one another. The sample includes mothers from the West (n = 5), South (n = 2), Northeast (n = 2), and Midwest (n = 1) regions of the U.S. and from rural (n = 3), urban (n = 3), and suburban (n = 4) communities. Parents had between 1 and 6 children ranging from 2 to 26 years old. See Table 1 for more demographic information about the sample.

**Table 1:** Demographic information for analytic sample

Participant pseudonym	ER identification of interviewed parent^1^	ER identification of children	Number of children	Age and gender of children	Geographic region	Urbanicity	Subjective social standing^2^	Occupation
Emma	White	Black/white	1	7F	South	Rural	3	Caretaker
Jia	Asian (Chinese)	Asian (Chinese) /Latino (Mexican)	3	13 M, 19F, 24 M	West	Urban	6–7	Bookkeeper
Chesa	Asian (Filipino)	Asian (Filipino) /white	3	16F, 22F, 24 M	West	Suburban	8	Interior designer
Samantha	White	Native American/white	2	2F, 6 M	Northeast	Rural	4	Personal care assistant
Ava	Native American	Native American/Latino (Mexican)	7	Deceased, 2 M, 12 M, 15F, 17F, 19F, 21F	West	Urban	6	health aid
Clara	Native American	Native American/Black	2	6F, 17 M	Midwest	Urban	10	Stay-at-home parent
Althea	Asian (Filipino)	Asian (Filipino) /Latino (Cuban)	2	8 M, 12 M	West	Suburban	7	Real estate broker
Elena	Latino (Puerto Rican)	Latino (Puerto Rican)/Black (African American)	3	16F, 21 M, 26F	South	Rural	3	Unemployed
Tala	Asian (Filipino)	Asian (Filipino) /white	2	12F, 14 M	West	Suburban	5	Elementary school Paraprofessional
Julia	Black (Caribbean American)	Black (Caribbean American) /white	2	2 M, 4 M	Northeast	Suburban	8–9	Lawyer

^1^ Self-described Ethnic-racial identity disclosed during the interview

^2^ See also; Adler, N. E., Epel, E. S., Castellazzo, G., & Ickovics, J. R. (2000). Relationship of subjective and objective social status with psychological and physiological functioning: Preliminary data in healthy, White women. *Health Psychology, 19*(6), 586–592. https://doi.org/10.1037/0278-6133.19.6.586; Diemer, M. A., Mistry, R. S., Wadsworth, M. E., López, I., & Reimers, F. (2013). Best Practices in Conceptualizing and Measuring Social Class in Psychological Research. *Analyses of Social Issues and Public Policy*, *13*(1), 77–113. https://doi.org/10.1111/asap.12001

### Interview Protocol and Procedure

Semi-structured virtual interviews lasting approximately one hour were conducted by three trained interviewers. Participants consented to having their interviews recorded and were compensated $100 for their time. We employed a semi-structured interview protocol aimed at gaining an understanding of how parents currently talked about racism and xenophobia in the home and what resources they thought would be helpful to further broach these topics with their children. First, demographic information was collected to better contextualize their experiences and learn more about their home and community contexts. Then participants were asked to elaborate on their own experiences with racism and xenophobia and those of their children. Parents were then prompted to share what content would be useful in facilitating conversations about racism and xenophobia with their children. Finally, we assessed how parents and children would logistically access content related to such conversations. Transcribed interviews were analyzed in Dedoose. This research was determined to be exempt from review by the University of Michigan IRB.

### Positionality

As an authorship team, we recognize that we share a commitment towards antiracism while acknowledging that we enter this work with different social identities and lived experiences which inform our own individual motivations, approaches, and understandings. Although we have each experienced being a member of a multiracial household, our individual lived experiences are different from each other, as we have occupied different spaces in this context. Below, we share how our individual positionalities inform how we approach antiracist work (see also Holmes, 2020; Milner, 2007).

VAV identifies as a biracial white-Latina woman. Her father is white and mother is Mexican American. She was born and raised in California and her parents did not talk about race in the home and often reinforced colorblind ideologies. VAV’s lived experience in this home context motivates her to do this work. It was not until young adulthood that VAV began to critically understand her own racial identity and racism more broadly. As a first-generation college student, VAV often finds herself sharing her knowledge about racism with her parents. VAV sees parallels between her own upbringing and some of those revealed in the transcripts in this study. She hopes that this work will encourage monoracial parents to become more comfortable with discussing race and racism in the home and work towards antiracism.

LAJ is a Korean American adoptee. She was born in Korea and raised in the American South in a white family. Although she was raised in a family that promoted colorblind ideologies and ignored the realities of racism, as an adult she recognizes that maintaining these ideologies and practices can cause and perpetuate harm. She holds the belief and personal experience that antiracism is always a choice, regardless of a person's background, and hopes that this work will support parents of multiracial households in having conversations with their children that help them to navigate the world in antiracist ways.

DRD is a Latina child of working-class immigrants who is now raising children with a white, non-Latino partner. DRD aims to work in solidarity with other people of color who experience various intersectional oppressions that do and do not mirror her own to continuously advance our collective emancipatory agency. She believes antiracism socialization is a daily practice, which she endeavors to engage with her own children every day.

### Coding and Analysis Approach

The research team adopted a consensual qualitative analysis approach (Hill et al., 1997). Preliminary analysis of the data included a start list of domain codes informed by Renn’s (2003) and Miville’s (2005) conceptualizations of the microsystem, CRT (Solórzano & Yosso, 2002), MultiCrit (Harris, 2016), and extant multiracial socialization (Csizmadia & Atkin, 2022) frameworks (see Table 2). The coders (VAV and LAJ) read through all ten transcripts in their entirety, memoing initial thoughts about insights and reactions prior to coding and analysis. They then conducted line-by-line coding of all the data to arrive at broader domains. The primary goal of the analysis was to assess multiracial RES and FSME socialization practices by identifying excerpts that either indicated a lived experience of the parent or child (Harris, 2016; Solorzano & Yosso, 2002), an RES or FSME socialization practice (Csizmadia & Atkin, 2022), a critical person socializing the child (Miville et al., 2005; Renn, 2003), or a critical place where the child was socialized (Miville et al., 2005; Renn, 2003), and then applied codes to these excerpts. Following the initial code application, coders convened and discussed the coded transcripts until they arrived at a consensus decision for each excerpt and assigned codes. This process included rigorous discussion, disagreement, adjustment, and consensus building related to the suitability of the assigned codes. The coding team repeated this method until all transcripts were analyzed.

**Table 2:** Codebook used in analyses

Parent code	Child code	Definition
MultiCRT	MCRT-ahistoricism	“Challenge to ahistoricism: in order to more fully understand the experiences of multiracial students, this tenet can be used to historically analyze specific issues, such as the addition of the ‘check all that apply’ option on the US census and college admission applications. (Harris, 2016, p. 800)”
MultiCRT	MCRT-intconv	“Interest convergence: this tenet exposes how multiracial students, who are positioned as objects to market diversity, are acknowledged only when it benefits the needs of the white institution. (Harris, 2016, p. 800)”
MultiCRT	MCRT-expknow	“Experiential knowledge: exploring the experiential knowledge of multiracial students centers their voices as well as challenges dominant ideologies concerning race and multiraciality.” (Harris, 2016, p.800)
MultiCRT	MCRT-domideo	“Challenge to dominant ideology: dominant ideologies are challenged when narrative voice is utilized and the experiences of multiracial students are foregrounded in research.” (Harris, 2016, p.800)
	MCRT-domideoREINFORCE	Perpetuates or reinforces dominant ideology
	MCRT-domideoRESIST	Resists or challenges dominant ideology
MultiCRT	MCRT-colorism	“Racism, monoracism, and colorism: this tenet accounts for multiracial students’ encounters with racism, as well as monoracism and colorism.” (Harris, 2016, p.800)
MultiCRT	MCRT-mono	“A monoracial paradigm of race: to fully account for multiracial students’ racialized experiences, the concept of a monoracial paradigm of race expands beyond a critique of the black/white binary and focuses on the way that race is constructed in neat, fixed categories, disallowing for the recognition of a multiracial reality.” (Harris, 2016, p. 800)
MultiCRT	MCRT-microrac	“Differential micro-racialization: this tenant accounts for the timing of differential racialization. Multiracial students are racialized differently on a daily basis to serve the needs of the white institution.” (Harris, 2016, p. 800)
MultiCRT	MCRT-intersect	“Intersections of multiple racial identities: this tenet allows for an exploration beyond the intersection of singular social identities and examines the intersections of multiracial students’ racial heritage(s).” (Harris, 2016, p.800)
Racial identity development	RID-critperson	Relationships with parents, extended family, friends. (Miville et a. 2005; Renn, 2003)
	CritPARENT	Refers to a relationship between child and parent or grandparent. (Miville et al. 2005; Renn, 2003)
	CritPEER	Refers to a relationship between child and peers/friends. (Miville et al. 2005; Renn, 2003)
	CritMEDIA	Refers to a relationship between child and media source
	CritOTHER	Refers to a relationship between child and another critical adult in their life (teacher, coach, etc.)
Racial identity development	RID-critplace	Settings of growing up, such as school. (Miville et al. 2005; Renn, 2003)
	LVD-Home	Refers to an event or experience that took place in the home or in a family setting
	LVD-Community	Refers to an event or experience that took place in the community (including school, work, etc.)
MRSupport	RES-culturalsoc	Cultural socialization: “can involve implicit and explicit messages aimed to install racial-ethnic and cultural pride, engagement in traditions and activities designed to celebrate and maintain familial racial-ethnic and cultural artifacts.” (Csizmadia & Atkin, 2022, p.669)
MRSupport	RES-prepbias	Preparation for bias: “familial messages and activities aimed at helping children anticipate racial bias and equipping them with skills to cope with racial discrimination, is protective in nature.” (Csizmadia & Atkin, 2022, p. 669)
MRSupport	RES-mistrust	Promotion of mistrust messages: “aim to inculcate wariness towards racial outgroup members without explicit instruction on how to cope with racial bias.” (Csizmadia & Atkin, 2022, p.669)
MRSupport	RES-neg	Negative socialization: “messages that teach children prejudicial attitudes towards a racial group, including one of their own ingroups; also includes invalidating or discriminatory messages from parents to their own children” (Csizmadia & Atkin, 2022, p. 670)
MRSupport	RES-egal	“Messages that promote racial equality, and often colorblindness” (Csizmadia & Atkin, 2022, p. 670)
MRSupport	RES-race conscious	Race-conscious socialization: “messages that teach youth to treat everyone equally while acknowledging systemic racism and racial inequality” (Csizmadia & Atkin, 2022, p.670)
MRSupport	RES-diversity	Diversity appreciation socialization: “does not address racism, but teaches youth to respect people of different racial-ethnic backgrounds and appreciate their cultural differences.” (Csizmadia & Atkin, 2022, p.670)
MRSupport	RES-colorblind	Colorblind socialization: “messages that convey that race does not matter and everyone is the same.” (Csizmadia & Atkin, 2022, p.670)
MRSupport	RES-silent	Silent socialization: “they do not have explicit conversations or avoid discussions about race and race-related issues” (Csizmadia & Atkin, 2022, p.670)
MRSupport	RES-exposure	Exposure to diversity socialization: “a nonverbal type of socialization that involves parents exposing their children to racially diverse context.” (Csizmadia & Atkin, 2022, p.670)
MRSupport	RES-multi	Multiracial identity socialization: “messages from parents about their children's multiracial identity” (Csizmadia & Atkin, 2022, p. 670)
MRSupport	FSME-multiconsc	“Multracial conscious support openly acknowledges multiraciality as a unique experience and identity.” (Csizmadia & Atkin, 2022, p.671)
MRSupport	FSME-multiheri	Multiple heritage validation: “parents support youths belonging to multiple monoracial groups.” (Csizmadia & Atkin, 2022; p.671)
MRSupport	FSME-connect	Connection support: “affirmations from parents that addressed distress over not feeling connected to parents, either because multiracial youth did not physically resemble them or because they did not know enough about their culture.” (Csizmadia & Atkin, 2022, p.671)
MRSupport	FSME-racialdis	“Racial discrimination support: parents validating their child's discrimination experiences, related to or empathizing, giving advice on responses to discrimination for their children.” (Csizmadia & Atkin, 2022)
MRSupport	FSME-multiexpression	“Multiracial identity expression support: parents being supportive and encouraging youth to explore their multiple heritages and identify with any or all groups as they wished.” (Csizmadia & Atkin, 2022)
CERI		Structural Code: Parent describes the ethnic-racial identity of their child/children
Critical race theory	CRT-intercen	“The intercentricity of race and racism with other forms of subordination. CRT starts from the premise that race and racism are central, endemic, permanent and a fundamental part of defining and explaining how US society functions (Bell, 1992; Russell, 1992). CRT acknowledges the inextricable layers of racialized subordination based on gender, class, immigration status, surname, phenotype, accent and sexuality (Crenshaw, 1989, 1993).” (Solórzano & Yosso, 2002, pp. 25–26)
Critical race theory	CRT-domiedo	“The challenge to dominant ideology. CRT challenges white privilege and refutes the claims that educational institutions make toward objectivity, meritocracy, color-blindness, race neutrality and equal opportunity. CRT challenges notions of ‘neutral’ research or ‘objective’ researchers and exposes deficit-informed research that silences, ignores and distorts epistemologies of People of Color (Delgado Bernal 1998). CRT argues that these traditional claims act as a camouflage for the self-interest, power, and privilege of dominant groups in US society (Bell, 1987; Calmore, 1992; Solórzano, 1997).” (Solórzano & Yosso, 2002, p. 26)
	CRT-domideoREINFORCE	Perpetuates or reinforces dominant ideology
	CRT-domideoRESIST	Resists or challenges dominant ideology
Critical race theory	CRT-socjust	“The commitment to social justice. CRT is committed to social justice and offers a liberatory or transformative response to racial, gender and class oppression (Matsuda, 1991). Such a social justice research agenda exposes the ‘interest-convergence’ (Bell, 1987) of civil rights ‘gains’ in education and works toward the elimination of racism, sexism and poverty, as well as the empowerment of People of Color and other subordinated groups (Solórzano & Delgado Bernal, 2001).” (Solórzano & Yosso, 2002, p. 26)
Critical race theory	CRT-expknow	“The centrality of experiential knowledge. CRT recognizes that the experiential knowledge of People of Color is legitimate, appropriate, and critical to under- standing, analyzing and teaching about racial subordination. CRT draws explicitly on the lived experiences of People of Color by including such methods as storytelling, family histories, biographies, scenarios, parables, cuentos, testimonios, chronicles and narratives (Bell, 1987, 1992, 1996; Delgado, 1989, 1993, 1995a, b, 1996; Espinoza, 1990; Olivas, 1990; Montoya, 1994; Carrasco, 1996; Solórzano & Yosso, 2000, 2001; Solórzano & Delgado Bernal, 2001).” (Solórzano & Yosso, 2002, p. 26)
Critical race theory	CRT-transdis	“The transdisciplinary perspective. CRT goes beyond disciplinary boundaries to analyze race and racism within both historical and contemporary contexts, drawing on scholarship from ethnic studies, women’s studies, sociology, history, law, psychology, film, theater and other fields (Delgado, 1984, 1992; Olivas, 1990; Harris, 1994; Garcia, 1995).” (Solórzano & Yosso, 2002, pp. 26–27)

Once all transcripts were coded, the research team met together to construct core ideas (Hill et al., 1997) from each participant. This process involved creating whole-case summaries of each participant and an abstraction of patterned applications of individual codes. Following the construction of core ideas, the research team engaged in a cross-case analysis by looking across individual cases and clustering core ideas into categories. This cross-case analysis was conducted through an investigation of the most frequent code co-occurrences. Subsequently, the research team generated a conceptual heuristic tool to describe how parents were reinforcing or resisting dominant racial ideologies and then charted different socialization practices onto this spectrum to create core categories. For example, reinforcing dominant ideologies co-occurred frequently with socialization practices that promoted color evasion, egalitarian ideas, and silence surrounding discussions of race or racism. The research team then identified which socialization practices were general, typical, and variant to the sample, and these differences in core ideas were mapped onto the heuristic spectrum of parent perspectives of dominant racial ideologies. This process resulted in the identification of five qualitatively different approaches to antiracist socialization.

Exemplary transcript excerpts were then identified for each socialization approach to better convey how these practices were enacted.

## Findings

**Fig. 1:**
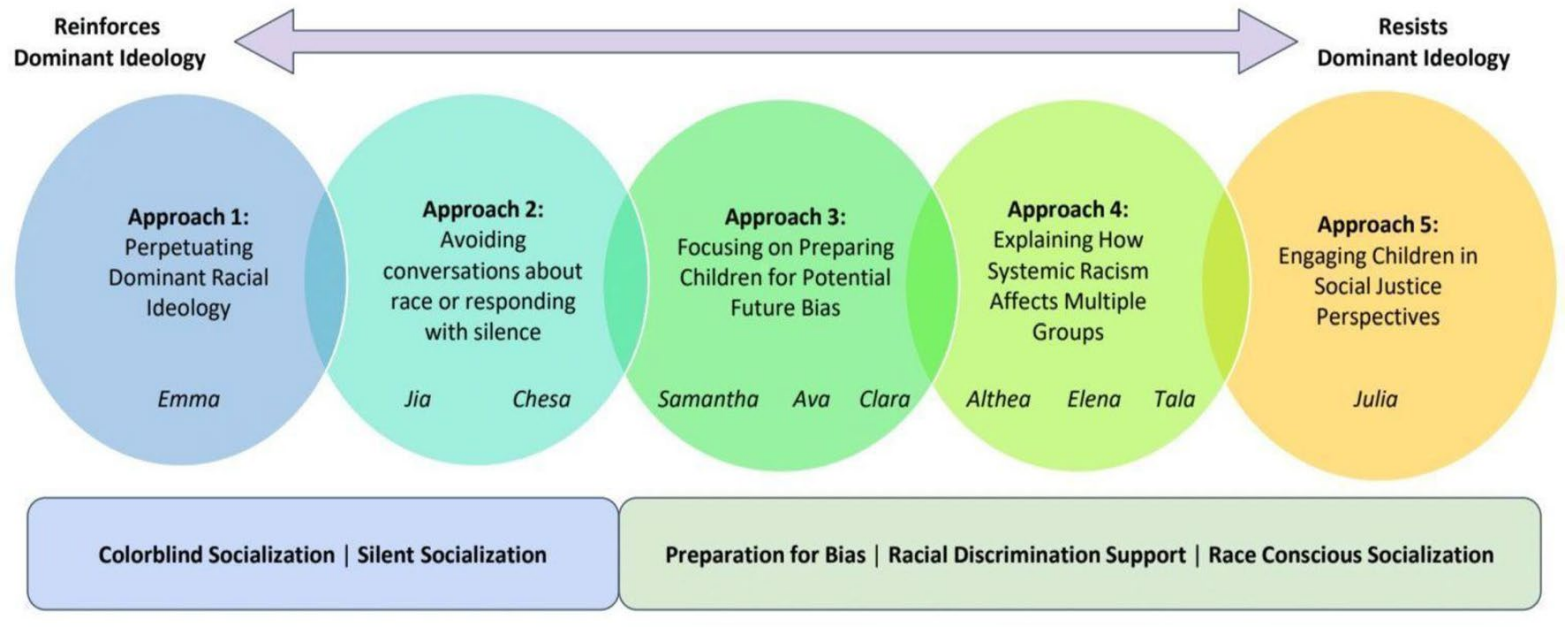
Five approaches to multiracial socialization that serve to resist or reinforce dominant ideologies

In this study, monoracial parents of multiracial children evinced five different approaches to socializing their children about race, racism, and antiracism (see Fig. 1). Important to the discussion of parents’ socialization practices is the general finding that all parents in the sample expressed in some way a recognition that their children’s experiences with race and racism are different from their own and in doing so acknowledge that their children’s multiracial identities are unique. Although parents adopted different approaches, ranging from those that perpetuate racism to those that promote antiracism, each parent’s decisions are grounded in care and love for their child and a belief that they are best preparing them to navigate the world as a multiracial individual.

We found that parents in our sample who heavily relied on color-evasive and silent socialization practices (approaches 1 and 2) typically reinforced racism and perpetuated the ideology that racism is not a real problem in their children’s lives or in society. By contrast, those parents in our sample who prepared their children for potential bias, validated and supported their children’s’ lived experiences with racism, and engaged in race-conscious socialization (approaches 3, 4, and 5) were better positioned to resist and disrupt racism and promote an antiracist orientation in their home.

### Approach 1: Perpetuating Dominant Racial Ideology

Some parents implement socialization practices in the home that reinforce dominant racial ideologies by taking a color-evasive approach that “deemphasizes the social importance of race” and denies the realities of racism (Csizmadia & Atkin, 2022, p. 670). Approaches that minimize racial differences also have the potential to ignore or erase children’s lived experiences with racism. One participant in our sample exemplifies how monoracial parents of multiracial children can maintain racist ideologies and inadvertently perpetuate these ideas onto her child, despite the racialized concerns she has for her child’s development.

#### Emma^3^

Emma is a white mother of a 7-year-old biracial Black/white daughter in the rural South. Early in the interview, Emma noted that she does not like to talk about racism with her Black husband because it “can get awkward.” Her discomfort around talking about race and her belief that racism is not real create challenges for her in supporting her biracial Black/white daughter. When asked to elaborate on her ideas about race and racism, Emma responded:

To me it feels like the government, the higher powers, the media want to divide people and so they make race a much bigger deal than it is. When I say something like that to a majority of my friends that are African American, they think I'm crazy and don't know what I'm talking about, but then I do have some friends that totally agree with me. And they feel like if the government would quit labeling us, it would bring us together. But I think that they do that on purpose to keep us divided, to keep us distracted from all the horrible things they're doing. Because if they've got the different groups of people fighting amongst each other, they're exploring all the crimes that everybody else is getting away with. I wish everybody would just treat each other equally and we're all just a different shade of basically the same color.

Emma believes that the government and the media have unnecessarily foregrounded race and racism as a problem in America, and that this focus on race and racism has created a divisiveness and hostility that otherwise would not exist. Emma’s belief that the government and media have manufactured racism as an issue in America upholds the dominant racial ideology that racism is an invented problem and denies “the existence of racial inequality” (Bonilla-Silva, 2015, p. 1361). Although Emma acknowledges that there are “horrible things” happening in our society, she believes that mentioning race is a distraction from societal change. Throughout her interview, Emma maintains this color-evasive ideology where she “deemphasizes the social importance of race” (Csizmadia & Atkin, 2022, p. 670) stating that she “would like to see people no longer referred to as Black, White, we're American.” Emma prefers to ignore racial differences and the social realities of racism. Emma identifies *talking about racism* as the problem rather than racism itself, and this belief influences how she chooses to engage her child in conversations about race.

Throughout the interview, Emma repeatedly asserts that racism is an invented issue and often positions people of color as the perpetrators of racism. Additionally, Emma is unreflective on her stance and expressed disinterest in learning more about perspectives different from her own. During the interview, Emma remarked,

I feel like I come across as insensitive to some people because I deny that there's as much of an issue and a lot of people they're like, well it's because you haven't lived in my shoes, blah blah. I'm like, well quit that.

She believes that because she and her husband have not experienced racism in the form of others “burning our house down or doing anything crazy–that's what I would think of as racism is when you are targeted just for your race” that racism does not exist.

When asked what racism looks like in her daughter’s everyday life, Emma inadvertently perpetuates her investment in the dominant racial ideology through her color-evasive socialization of her biracial Black/white daughter. When asked what racism looks like in her daughter’s life, Emma replied,

I don't think she knows very much about racism at all…Because I try to teach her. I know she will eventually experience it and hear about it, but I want her formative years [for] her to be taught it doesn't matter the color of your skin. Everybody's a different color. And you can't tell if somebody's good or bad just based on their skin color, you've got to get to know them…She doesn't know much about race, I don't think. Well, I mean, we've touched on it just a little bit, because I don't want her to feel bad or feel like being the shade that she is different or bad, or that I don't want her to think that anybody is better based on their skin color. So I've really tried to teach her that.

Although Emma denies racism exists, she does paradoxically express concern and certainty that her daughter will eventually experience racism. Her concern for her daughter is the only time that Emma acknowledges a racial reality, and she immediately shifts from acknowledging this racial reality to emphasizing color-evasive egalitarianism with her daughter. Emma wants to prepare her daughter for future experiences with racism, but her desire conflicts with her belief that racism is an invented issue. She chooses to socialize her daughter by sending “messages that convey that race does not matter and everyone is the same” (Csizmadia & Atkin, 2022, p. 670), and in doing so, Emma ignores the reality that although race should not matter, it often does. By ignoring this reality, Emma is not preparing her daughter for potential future bias and racist experiences. Although this parent may be perpetuating racist ideas, she still shows concern for the wellbeing of her multiracial child, fearing that her children may be racialized in the future and that she may not be able to adequately support her.

### Approach 2: Avoiding Conversations about Race or Responding with Silence

Avoidance and silence are two socialization practices that involve parents not addressing issues of race, racism, immigration, or xenophobia. Parents in our sample who engaged with these socialization practices avoided these topics altogether or responded with silence when their children had questions about race or experiences with racism. Different from Emma who emphasized egalitarianism and downplayed the realities of racism in approach 1, Jia and Chesa do not refute the existence of racism. However, they do not create opportunities for children to understand their experiences with racism, nor do they provide support to children for responding to racism in their own lives. Additionally, parents in our sample who engaged in approach 2 also communicated that experiences with racism are individual, interpersonal interactions and did not recognize racism as a systemic issue. Often this silent approach was coupled with a belief that children have not experienced racism already or that children will not experience racism in the future. Often, these parents trusted that schools, peers, and community resources would educate their children about race.

#### Jia

Jia is an Asian (Chinese) mother from the urban West with a multiracial family with two adult children and a 13-year old biracial Asian/Latino (Chinese/Mexican) son. In her interview, Jia was hesitant to address race directly, circumventing questions so that she could discuss issues that were race neutral. After some prompting, Jia eventually shared a few experiences from her own childhood where she experienced discrimination both for her language and for her race. When asked if she ever talked with her parents about her own experiences or her parents’ experiences as immigrants, Jia responded, “Not really. No…I guess growing up I didn't really pay much attention to it.” Jia seems to have been socialized with racial silence in her own childhood, and she now socializes her children in this same way. Her silence is most noticeable when she briefly discusses her youngest son’s “biological father [who] got deported when he was about four…So we talked about it then, and then every year we would go to Mexico to visit him and his extended family there.” Jia has taken her son to visit his father for “about 7–10 days” every year since he was deported to Mexico. Her decision to do this demonstrates a high priority for supporting the relationship between her son and his father and also a commitment to culturally socializing her son as she “maintains familial racial-ethnic and cultural heritage” (Csizmadia & Atkin, 2022, p. 669). Although Jia helps her son to maintain his connection with his father, her conversations with her son focus on family relationships and are absent of larger discussion about race and immigration. When asked if her son had ever asked questions about “immigration or his ethnic-racial identity,” Jia provided her son with library books that “answered a lot of questions when he was little…So that covered a lot of it.” Although Jia helped her son to find these resources, rather than using these books as a starting point for conversation about their family’s situation, she relied on these resources alone to support her son in understanding the general topic of immigration.

#### Chesa

Chesa is an Asian (Filipino) mother from the suburban Northwest with two adult children and one 16-year old daughter, all of whom are biracial Asian/white (Filipino/white). Chesa shares how she has experienced racism in her current home community, and she describes these encounters as “weird.” As she further reflects on these encounters, she regrets that she did not respond to her recognition of racism in the moment; she says, “I should have said something. And that's what I was angry about.” Chesa is still learning to recognize and respond to racism in her own life separate from her children, and her silent reflections and dismissiveness of racism as “weird” manifests in her silent socialization about race with her children.

Chesa shares that her daughter’s friend group is mostly girls who are biracial Asian/white. However, she explains that this happened by coincidence, not acknowledging the possibility that her daughter could have self-selected these friends due to their shared biracial identity. Chesa’s teenage daughter is highly involved in school activities as the president of her class at school and is also engaged in organizing and activist work in the community. For example, Chesa’s daughters participated with their friends in a Black Lives Matter protest. Although Chesa and her husband eventually supported their daughter in attending the protest, Chesa describes how they reached this decision together:

And so the parade was a little, I mean, it was scary, right? … [When my daughters wanted to participate in a Black Lives Matter protest,] the mom part of me was like, “No, I don't want you to go, right.?” It sounded too dangerous, but they both talked to me and said, “You know what, mom this is the information that [another parent] has. I feel I need to be a part of this movement.” And it was so passionate and so loving and supporting that I just couldn't say no, I just couldn't. So now that I knew that it was safe and I was okay with it…. Because all the events that led up to this protest, they were terrible. They were--people were being jailed. People were hurt. People had bombs, people had firearms, people had gas tear it was bad. So and this one felt and looked and was marketed differently. But I'm so glad that they were part of it. And they both said, “I'm so glad I was a part of that.” I didn't do that and maybe that was because I didn't have an opportunity to, right? Nothing surfaced during my lifetime. But it's kind of that feeling when I was telling you, I should have said something at that dinner. They felt they needed to do this. And I remember that feeling and I'm like, yeah. Okay.

Throughout this story, Chesa shares that her daughters were passionate about attending the Black Lives Matter protest because they were interested in “supporting their friends.” Rather than engaging with her daughters about their passion for racial justice, Chesa chooses instead to focus on the logistics of the protest, and her focus on fear and safety perpetuates a dominant belief that Black Lives Matter protests are violent and dangerous. After the protest, Chesa reflects on how important attending the protest was for her daughters, and she directly connects this back to her own silence. Additionally, Chesa reflects, “I think as parents sometimes we just don't get it, and I'm going to be first to say that, right? We don't get what they're going through because it feels such a different time.” Although Chesa takes a silent approach to engaging with racism and socializing her children to be antiracist, she acknowledges that racism is real and does not prevent her children from pursuing their own antiracist learning or from engaging in antiracist activism in a way that is meaningful to them.

Chesa and Jia both have concerns about their children’s wellbeing. Chesa is hesitant for her daughter to attend a protest due to her safety and is excited that she has a group of close girl friends that share her values, and coincidentally, her biracial Asian/white identity. Jia makes a concerted effort to maintain a connection between her son and his father who lives in Mexico. Although racialized issues are salient in both of their homes, Chesa and Jia both avoid conversations about race or respond to their children’s racialized concerns with silence. These parents are both aware that interpersonal racism exists, an understanding that was partially formed from their own lived experiences with discrimination. However, they do not see the immediate relevance for their children and therefore do not discuss it at home. Because conversations about race and racism are avoided, their children must work to develop an antiracist orientation on their own, drawing knowledge and resources from peers, teachers, social media, or other sources of information.

### Approach 3: Focusing on Preparing Children for Potential Future Bias

Other parents in our sample began to take an antiracist approach by emphasizing the preparation for future bias for their children, often focusing on how children might experience racism based on one of their parents’ ethnic-racial identities. Different from parents who took a color-evasive or silent approach (approaches 1 and 2), this approach to antiracism includes parents in our sample who were having conversations with children about their multiple heritages. However, these conversations typically focused more on the ethnic-racial identity or heritage of one parent more than the other. This monoracial focus socialized the child to understand this part of their identity either as a point of pride and cultural learning or as a source of fear, danger, and shame. Although these socialization practices are “protective in nature” (Csizmadia & Atkin, 2022, p. 670), without a larger critique of systemic racism, these parents may inadvertently communicate to their children that part of their multiracial identity is a negative quality that they must be prepared to live with. Three mothers in our sample–Samantha, Ava, and Clara–engaged in this approach; they acknowledged racism as a real issue and were concerned about how it may affect their children. As a result, they engaged in conversations with their multiracial kids to prepare them for future potential bias. However, they think of racism as interpersonal and do not address racist systems or how racism may impact other ethnic-racial groups not represented in the home.

#### Samantha

Samantha is a white mother to two biracial Native American/white children, a 6-year old son and a 2-year old daughter, in the rural Northeast. In describing her multiracial family and the context in which they live, Samantha explains that her partner’s tribe determines tribal affiliation matrilineally. Within their community, Samantha explains that her children are “considered white, even though they're mostly Native American.” Samantha wants to prepare her children for future bias they might experience in their Native American community for having a white mother. Since tribal affiliation is determined matrilineally, her children are not considered a part of their father’s tribe. She explains that “I understand completely in wanting to keep their blood pure and their traditions alive but it also creates a lot of racism against people who are half and half or don't have a clan.”

Beyond their immediate community, Samantha also wants to prepare her children for future bias they might experience for being “half-white and half-Native American.” She draws on the cultural strengths from both of their monoracial backgrounds in order to prepare her children for the bias they might experience for being perceived only as white or only as Native American. For example, Samantha and her partner “talked to [our children] about where our ancestors came from… and how they have both [European and Native American] in their blood and how that makes them twice as strong.” Although Samantha teaches her children about “the positives about both sides” of their heritage, she emphasizes pride in their Native American heritage and expresses most of her fear of discrimination around their white identity. She did not mention concern about racism as it applies to other ethnic-racial groups not represented in the home and was only interested in talking about interpersonal racism as it applied to her children.

#### Ava

Ava is a Native American mother of seven biracial Native American/Latino (Native American/Mexican) children, ages 2 to 21, in the urban West. Ava shares that she has been learning “that I just have to accept everybody…Maybe before I didn't always, because I was closed-minded.” She attributes her learning to her 17-year old daughter who initiates conversations about race, immigration, class, gender, sexuality, and religion at home and challenges the family to take a social justice stance. Ava provided several examples where her daughter created a formal presentation to educate her parents and then invited them to have a discussion. Ava shared that her own beliefs are changing as a result of these conversations to align more with her daughter but that her husband maintains his “closed-minded” opinions. Based on the examples she shares, her husband reinforces the dominant ideology at home by promoting racist, xenophobia, homophobic, and Islamophobic views. Although Ava’s husband maintains these ideologies and vocalizes them in their home, their children nevertheless choose to initiate these conversations with their parents with the hopes that they can foster a greater social justice orientation in their family. Conversations about race and racism in this home are not taboo topics and although Ava does not actively engage in race-conscious socialization, she is proud to see her daughter engaging in this discourse and raising these issues within the home to challenge her own thinking.

Ava also privately expresses variable concerns for her multiracial children as informed by their phenotypes. For instance, she describes her 15-year old daughter as “pale, fair skinned” and blonde. She expresses concern about her appearance because her daughter has been teased and ostracized at school for not being Native American or Mexican enough. She says that her daughter’s peers “don’t make fun of her, but talk crap to her about the color of her skin.” Additionally, Ava’s middle school-aged son has darker skin, and she fears he may be profiled and brutalized by police due to his appearance. She explains.

Because he looks African American–once again it's genetics. And there's a lot happening with African American boys in the police. So he's not allowed to go out after hours, he mainly stays home…So that's a fear for me and my husband, and we have told him if a police officer stops him, you just lift your hands up and you don't don't do nothing they don't tell you not to do. And it's sad that we have to tell him that, because he's only 12.

In addition to these conversations with her son, she also does some self-learning to better understand what he may experience. Ava explains that she does Google searches and joined a Facebook group that talks about textured hair care to get advice and guidance on how to support her dark-skinned son. She says the group interests her because they talk about more than just hair care. She explains that parents ask a variety of questions like,

Is this going to make my son look gang affiliated? I mean, it's just stupid little questions that shouldn't matter, but to parents, these things matter, they don't want their kids hurt for their hairstyle. They don't want them robbed for their shoes. So to us the questions seem...This could happen to our kids.

Ava acknowledges the variability in her multiracial children’s lived experiences due to their diverse phenotypes and acknowledges interpersonal racism and attempts to prepare her children for racial bias while drawing knowledge from other parents to learn how best to approach these conversations with her kids.

#### Clara

Clara is a Native American mother of two biracial Native American/Black children, a 17-year-old son and a 6-year old daughter, in the urban Midwest. Clara is a single mother who wants to learn how to talk to her children about anti-Black racism and understands that her own lived experience has not prepared her to have these conversations. Clara repeatedly notes how her own experiences differ from her children’s: “I could totally pass as white. Nobody ever would even think I'm Native American unless I show my tribal ID, so I've never really had to experience racism.” Clara validates her children’s experiences with racial discrimination; however, she would like more support in helping her children in knowing how to respond or take action when they experience racism in their own lives. As she works to learn more, her current socialization strategy is to prepare her children for bias. During the interview, Clara stressed the importance of sharing a specific story about her son. She recounts:

My son was pulled over…He explained [it] to me, which almost brought me and my family to tears because–I would never think if I got pulled over for speeding, and they came to the window to me, and asked for my driver's license–I would turn over and be digging in my purse to get that driver's license, not even thinking. My son told me when he was pulled over because he's Black, he had his insurance card out the window before the cop came, and he had his one hand on the steering wheel, and he also turned on his dome lights. When the officer came to the window and asked, of course, for his ID, it was in his front coat pocket, on his chest, there was a little zipper. He had actually asked the police–when the police asked for his driver's license–he said, “It's in my chest pocket. Can I grab it?”…That almost brought us to tears because I would never think to have to do…I would just automatically go to my purse and start digging around…Probably from the age of 11, I've always told him they're going to look at him different. He's going to be viewed as Black, and unfortunately society puts a fear to that…I told him from a young age, “When you get older, even though you're such a good person, and we all know you're a good person, other people might not view you–they might view you as a threat because you have braids, because you're darker skinned, you're taller.” Just everything that has went on in the last few years with police and stuff like that, I just brought that to his attention. I said, “I'm sorry bud, but that's how it is. If you do get pulled over, you just remember to keep your hands where they can see them. You listen. Just do what they ask. It might not seem fair at the time, but I want you to be able to come home at night.”

Clara’s grief and fear of the reality of how her son experiences racial discrimination is coupled with an aching acknowledgment that she must prepare him for racism that she herself has never experienced. Rather than socializing her son with a critique of systemic racism, she focuses on preparing her son for how to interact in individual moments where he might be targeted. Her attention to how others will view him as Black is intended as protective, however, she may also be unintentionally communicating to her son that something about him being viewed as Black has the potential to coincide with him being viewed as dangerous. Because she does not have contact with either of her children’s fathers, she feels ill prepared to teach them about their heritage and cultural traditions. With the absence of positive cultural socialization, these messages that prepare her children for anti-Black bias may communicate to them that their Blackness is a liability.

Samantha, Ava, and Clara all expressed concern for the wellbeing of their children, especially as they navigate a racialized world where they may experience racial discrimination. As a result, these parents all engaged in conversations that serve to prepare their children for different forms of bias. They recognized that one or more of their ethnic-racial identities may influence how others perceive them and they are working to make sure that their children know how to respond in these situations. In a poignant example, Clara did so by explicitly giving her son instructions on how to interact with police as a Black male and empathizing with his recent encounter with law enforcement. This was particularly challenging for her because she cannot draw from her own lived experiences, nor the experiences of her children’s fathers. Nonetheless, she engaged her children in these difficult conversations out of concern for their safety and wellbeing.

Ava expressed different concerns for her multiracial children depending on how they phenotypically present. For her darker-skinned son, she worried he might be racially profiled by police, and she has done some internet exploration so she can better understand what her son may experience. Ava was nervous, too, that her white-passing daughter may feel ostracized by her peers for being neither Native American nor Mexican enough. Yet, Ava relies on her daughter to take a more active role in orienting the family to systemic social justice issues.

Samantha also shared concerns that her Native American and white children will be othered by their tribe due to their whiteness. Instead of overtly preparing them for bias in the way that Clara does with her Black son, Samantha draws on cultural strengths to instill racial pride in her children. She hopes that if her kids grow up knowing a lot about their heritage, they will be secure in their identity even when their authenticity is challenged. All three of these mothers center the needs and concerns of their children and prepare them for bias with different approaches. Collectively, they all understand that racism and other forms of bias exist and apply to their multiracial children and address them, to varying extents, at home. In these conversations, Samantha, Ava, and Clara are particularly concerned with how racism impacts the racial groups of which their children are a member, and they do not discuss how racism may impact people with other racial identities not represented in their home.

### Approach 4: Explaining How Systemic Racism Affects Multiple Groups

Socializing children towards antiracism involves supporting them in recognizing and responding to racial discrimination; three parents in our sample were actively doing this with their children. Racial discrimination support often involves parents empathizing with children through sharing personal lived experiences with racism and validating children’s experiences in race-conscious ways that acknowledge “systemic racism and racial inequality” (Csizmadia & Atkin, 2022, p. 670). Parents in our sample who used these practices involved their children in conversations about their multiraciality and larger conversations about ethnic-racial groups that were not represented in the home. Althea, Elena, and Tala drew directly on their own experiences with racism and sometimes transdisciplinary knowledge to inform their understanding of systemic racism. They shared this knowledge with their children, aiding their understanding of how systemic racism may impact various ethnic-racial groups. Additionally, these parents supported their children’s exploration and expression of their multiracial identities.

#### Althea

Althea is an Asian (Filipino) mother of two biracial Asian/Latino (Filipino/Cuban) sons, an 8-year old and a 12-year old, in the suburban West. Althea immigrated to the United States as a child, and she shared how she has experienced racism and xenophobia in both her childhood and adulthood. She feels that racism in her current everyday life is “not obvious…You may not see it upfront—but you could feel it. You could feel—it's kind of a hard thing to describe, but you can feel racism.” Althea believes that her children are “not really touched by [racism]…in a direct way,” and even though her children do not experience the same forms of overt racism that she did as a child, she still works to prepare her children for potential future bias. Her primary message to her children is that racism is a negative experience, but that there are many examples of people of color who have achieved great success “despite what happened to them.” Althea’s approach to socializing her biracial children focuses more on their shared Asian identity and how they navigate the world as individuals.

Although Althea focuses more on this interpersonal aspect of racism with her children, her socialization messages also attend to how individuals navigate racism as a result of racist systems. For example, when asked what racial justice issues are important to her, Althea responded, “I mean—there is always the problem with the police officers: how they treat people of different cultures and especially African Americans. But lately, because of COVID, how people–other people–are treating Asians.” Here Althea acknowledged that racism impacts other ethnic-racial groups not represented in the home. Furthermore, she drew from current events related to anti-Black racism when preparing her own kids for bias. For example, she explains:

Whenever something comes up in the news that looks like what they were doing—when people were breaking into the stores during those riots, we had to explain what was going on. And we're saying well, this is, this is the product of racism, and how they were—how they're treating other other races, and the police officers and all that. So it's there—there is racism, even if, even if you don't feel like yourself, and sooner or later, you're probably going to be affected by it directly. So, just know that no matter how people mean—if they, how well they mean to treat you, that there will be some judgment that will happen. Regard— even [if] it's your skin, or your, or your skin color, your religion, or whatever, it's going to happen because you're different.

In addition to these direct conversations that prepare her children for racial bias, Althea also expressed a desire to show them content of not just Asian role models, but also stories of other marginalized people (Latinx, Black people, immigrants, etc.) that have overcome discrimination. Althea conveyed a greater understanding of how racism impacts a variety of ethnic-racial groups and was keen to her kids about this.

#### Elena

Elena is a Latina (Puerto Rican) mother of three children–two adult-age and one 16-year old biracial Latino/Black (Puerto Rican/African American) daughter—from the rural South. Elena and her family have lived in many regional areas in the United States, most of them being rural, predominantly white communities; she and her children have experienced racism across each of these contexts. Elena recounted several instances where she personally experienced racial violence, and she draws from her own experiences as she supports her children in navigating racial discrimination and disrupting dominant racial ideologies such as white supremacy. Throughout her interview, Elena shares how she has conversations with her children that address colorism, language discrimination, gender discrimination, and ableism. These conversations are rooted in their family’s shared understanding that “The system is built for who it’s built for, and there isn’t enough going on right now, enough people that are actually in the system to change the system. And that’s a sad thing.”

Elena recounted how her daughter was racially profiled and stopped by police “because she was trying to walk home” after a sleepover. Drawing from her own experience being racially profiled and arrested in front of her son because she was presumed to be drunk driving, she was able to empathize with her daughter’s experience and offer her support. When Elena’s oldest daughter was in elementary school, she further modeled how to stand up to racism when she was being pushed into speech therapy and English as a second language courses due to their dual-language household status. Elena advocated for her daughter to be accommodated in mainstream courses.

Elena’s antiracist discussion and action with her children “acknowledges the intercentricity of racialized oppression–the layers of subordination based on race, gender, class, immigration status, surname, phenotype, accent, and sexuality” (Solórzano & Yosso, 2002, p. 25). The conversations that Elena has had with her children were often prompted by experiences in each of their lives, and she and her children endorse critical and activist perspectives on how racism manifests in education, healthcare, and criminal justice systems. Elena stated that she and her daughter often engage in conversations centering activism. Her daughter vocally addresses racism in online spaces, explicitly calling out anti-Blackness within the Latino community. For example, Elena shares how her oldest daughter watches gamers who livestream:

She called this guy out because he was doing--basically, it was blackfishing. It was a particular clip he’d put… on his YouTube channel. This child of mine, she knows that I protested and things like that. That’s how she’s so able to do it. She got that on his comments and stuff and went--had a full-on debate… Basically she’s like, “You know this? I’m Black… and I’m Latina, and I don’t appreciate this… You are dumb to your subscribers. This is insulting.”

Because Elena has been up front with her children about her own experiences with racism, her kids are better able to address and respond to racist incidents when they occur.

#### Tala

Tala is an Asian (Filipino) mother of two biracial Asian/white (Filipino/white) children, a 12-year old daughter and a 14-year old son, from the suburban West. Tala is specifically interested in racial justice issues such as Black Lives Matter, anti-Asian violence, and family separation at the U.S.-Mexico border. Although Tala mentions microaggressions in her workplace, she believes that the racism she has experienced is “not to the level” of her Black friends. In addition, in regard to her children’s experiences of racism in the form of colorism, Tala notes that her children have responded in ways that suggest pride in their Filipino and/or *hapa* (“mixed”) identities. For example, even when her son was an infant, strangers would approach her and ask “‘Who is that baby’s father?’… trying to figure out why my son’s so light.” As teenagers, her son is often asked “What are you?” to which he has begun responding that he is Filipino because that’s the “first thing people see when they see me.” Different from her son, her daughter “has a group of friends where they're all like, they have one white parent and one Asian parent,” and she more often identifies as “half.”

Tala studied Asian American Studies and sociology in college and shares her understanding of racism “within the historical context” with her children. For example, she socializes her children to understand how her interracial marriage and her children’s *hapa* identity fits within a larger historical context of interracial relationships in the United States, referring to *Loving v Virginia.* Within this larger historical context, Tala believes that “personal stories are really important” and socializes her children much in the same way that she taught an Asian American Studies course in graduate school, where “half of my lecture was just about personal experience–people asking me questions about personal experience [like] what it’s like to be Filipino in America.” She instills Filipino pride in her children but also acknowledges how racism applies to other ethnic-racial groups with an understanding that racism is rooted in history and systemic in nature.

In our sample, Tala, Elena, and Althea evinced a more robust understanding of race and racism in comparison to the parents described as socializing their children according to approaches 1–3. Their own lived experiences and transdisciplinary knowledge informed their understanding of systemic racism as it applies to multiple racial groups. This higher-level perspective on racism is evidenced in their race-conscious socialization practices with their children that not only center racism as it applies to their own multiracial identities, but also as it impacts other ethnic-racial groups not represented in their home. This messaging challenges dominant ideologies and is thus oriented toward antiracism. Notably, all of these parents stated that these conversations started to occur once their children were older. These mothers did not address systemic racism at home until they believed it was relevant for their children: as it was brought up in school, as they started having questions about their identity, or as the children themselves experienced racism. Although they are engaging in antiracist conversations that center racist systems broadly, these discussions started later in their children’s lives once they believed racism to be relevant to their children.

### Approach 5: Engaging Children in Social Justice Perspectives

A transformative approach to antiracist socialization requires that parents understand racism as a systemic issue with historical underpinnings. One parent in our sample was actively resisting dominant racial ideologies by intentionally socializing a social justice perspective with her children that considers their own multiracial identities and ethnic-racial identities that are not represented in the home. This work involves intentionally and critically socializing children to be race conscious and “to treat everyone equally while acknowledging systemic racism and racial inequality” (Csizmadia & Atkin, 2022, p. 670). In our sample, Julia best exemplified justice-oriented multiracial socialization, as she engaged in extensive self-learning and proactive, race-conscious socialization in order to better position her children to be what she calls “antiracist disruptors.”

#### Julia

Julia is a Black (Caribbean American) mother of two biracial Black/white (Caribbean American/white) sons, ages 2- and 4-years old, in the suburban Northeast. Julia shared that she finds antiracism to be “really exciting work” and that she has pursued learning about antiracism in both personal and professional contexts. When asked what racial justice means to her, Julia responded,

I would definitely say [racial justice looks like] opportunities for all, regardless of race. Really kind of removing some of the barriers that exist for groups that are disadvantaged, primarily on the basis of their race. I think about it really as kind of knocking down the roadblocks to opportunity, to acceptance for people, regardless of the way they racially identify…I'm thinking opportunities for employment, any type of discrimination that occurs as people try to get certain jobs or try to be promoted within a particular entity. I think about the criminal justice system and the ways in which it doesn't punish fairly around race. Since we talked about it too, I think about the immigration system, how I would say the United States doesn't treat immigrants who are, depending on the country they come from, fairly and justly…I think there's a number. I think that the one that comes to mind is–sorry I didn't mention it before–but violence on the basis of race. Just thinking about any uptick in white supremacist activity is particularly concerning to me. Any racially motivated violence, I feel like whenever I hear about it, it just makes me concerned for myself, my kids, and for the people that I know in my life, and even people I don't know, obviously.

Julia’s discussion of racial justice is linked to multiple systems including employment, criminal justice, and immigration, which demonstrates that she has an understanding of racial justice that “starts from the premise that race and racism are central, endemic, permanent and a fundamental part of defining and explaining how US society functions” (Yosso, 2005, p. 73). Her vision of racial justice aligns with Yosso’s (2005) conceptualization in that it is focused on eradicating oppression and empowering those who are affected by systems of oppression (Yosso, 2005). In contrast to Emma, who perpetuated dominant racial ideologies, Julia is an active resistor of such ideologies, and her antiracist orientation influences how she socializes her children. Her knowledge of these structures makes her fear for how her children may interact with this society, and so she is thinking critically and intentionally about how she will raise her children in a racist world from a very young age.

Julia provided several examples for how she has been intentional about introducing her young children to antiracist and activist ideas. When asked what else she might need to support her in talking about race, racism, immigration, and xenophobia with her children, Julia responded,

[I would like] ways to talk about race in an age-appropriate way that encourages my kids to be *antiracist disruptors*. It's important to be aware of race and not to be colorblind–but ways in which to sort of have those discussions with a four year old or with a five year old. Something that would bring in the developmental age and words to use to break that down…The [parent group that my husband attends] focuses more on advocacy, but I feel like I'm often kind of looking for almost a support group around how to help your kids navigate the world with an antiracist lens, and also how to prepare them to deal with any racism that they may face.

Julia differs from other parents in this sample in that she is socializing her children beyond race and racism as it relates to their own multiracial identities; she hopes to develop an antiracist orientation in her sons so that they can be disruptors of racial injustice. Her socialization practices reflect a commitment to social justice, and her practices with her young children offer a “transformative response” to different forms of oppression (Yosso, 2005, p. 74). Although all parents in this sample mentioned wanting to teach their children how to “counteract societal racial prejudice and bias,” Julia is one of the few parents whose goal is to “help youth be aware of the realities of racism that contribute to racial disparities” (Csizmadia & Atkin, 2022, p. 670).

Different from the other parents in this sample, Julia’s approach to antiracism is *proactive* rather than reactive; rather than responding to her children’s experiences with racism or preparing them for future encounters they may have directly, Julia views discussions about race, racism, and antiracism as important to how they view, experience, and navigate the world more generally. Not only does she want her to prepare her children to recognize racism, she wants to orient them to challenge racist ideologies and position them to be social justice activists. Even before her children have begun traditional schooling, Julia has purchased books that will help her sons learn about antiracism and activism and intentionally engages her children in these topics even though they are very young. She has done extensive self-learning as well–reading books and listening to podcasts that discuss how parents can engage their young children in conversations about racism. Additionally, Julia explains that she has already started talking to her kids about colorism, using language that they can understand.

Julia aims to go beyond preparing her children for bias and validating her children’s experiences with racism by critically and intentionally engaging her children in social justice conversations from a very young age, before they have even directly experienced racism for the first time. She draws from her lived experiences, transdisciplinary knowledge, and ongoing self-learning in socializing her children to be "antiracist disruptors." In her household, social justice issues applicable to other ethnic-racial groups not represented in her family are discussed with the same emphasis and importance of ethnic-racial issues relevant to the identities her children hold. Julia not only wants to prepare her children to respond to their own experiences with future bias, but also teach them to be race-conscious more broadly– recognizing all forms of bias. She hopes to motivate her multiracial children to recognize and dismantle dominant racial ideologies.

## Discussion

In this work, we map how multiracial socialization practices can either serve to resist or reinforce dominant ideologies in the home. Although all parents in our sample had an investment in their child’s wellbeing, some were socializing them in ways that perpetuate dominant ideologies and hinder their development of antiracist competencies. Our research articulates five approaches monoracial parents in our sample demonstrated when socializing their multiracial children around race and racism broadly. Parents were identified as either: (1) perpetuating dominant racial ideologies, (2) avoiding conversations about race or responding with silence, (3) focusing on preparing children for potential future bias, (4) explaining how systemic racism affects multiple groups, or (5) engaging children in social justice perspectives (See Table 3 for a detailed representation of these approaches and what they entailed).

**Table 3:** Moving through five approaches to antiracist socialization in multiracial households

Starting point	Approach 1 Perpetuating dominant racial ideology	Approach 2 Avoiding conversations about race or responding with silence	Approach 3 Focusing on preparing children for potential future bias	Approach 4 Explaining how systemic racism affects multiple groups	Approach 5 Engaging children in social justice perspectives
General Stance	Reinforces dominant ideology	May contradictorily both reinforce and resist dominant ideology		Resists dominant ideology	
Understandings of Racism in Everyday Life	Maintains either that racism is not real or that racism does not affect the everyday lives of their children		Acknowledges that racism is real and shares examples of racism from their own lives and from the everyday lives of their children		
	No emphasis on lived experiences with racism		High emphasis on children’s lived experiences with racism	High emphasis on own (parent) lived experiences with racism	Reflects on how children’s experiences with racism may differ from their own
	Non-reactive to experiences with racism		Reactive to family’s experiences with racism	Proactive about own and others’ experiences with racism	
Common Multiracial Socialization Practices (per Czimadia & Atkin, 2022)	High color-evasive socialization				
		High diversity socialization high egalitarian socialization		High exposure socialization	
		High silent socialization	High preparation for future bias	High racial discrimination support	High race-conscious socialization
Specific Socialization Practices	∙ Deemphasizes the importance of race ∙ Believes that racism is an invented issue	∙ Avoids conversations about race ∙ Believes that children do not and will not have experiences with racism	∙ Prepares children for potential future bias ∙ Focuses on ethnic-racial identities represented in the home ∙ Frames racism as mostly interpersonal rather than institutional or systemic	∙ Validates children’s experiences in race-conscious ways ∙ Engages children in conversations about ethnic-racial groups not represented in the home ∙ Supports children’s exploration and expression of their multiracial identities	∙ Understands racism as a systemic issue ∙ Connects racism through history into present day and contemporary issues ∙ Socializes children intentionally
Areas for Further Growth	∙ Acknowledging that racial difference exists ∙ Acknowledging that racism affects their children	∙ Responding to children’s questions about race and racism ∙ Preparing children for future bias	∙ Thinking about how racism affects multiple groups beyond those represented in the home ∙ Drawing connections and also recognizing differences between parent and child experiences ∙ Encouraging children to ask questions and offer their thoughts about race and racism at home	∙ Understanding that historical underpinnings of racism result in contemporary manifestations ∙ Recognizing what structural barriers still exist for various racial groups ∙ Being intentional about socializing children about race and racism from a very young age	∙ Continuing self-learning ∙ Finding opportunities for community engagement and activism ∙ Pursuing opportunities to do antiracist work as a family unit

The first two approaches reinforce dominant racial ideologies through the primary use of color-evasive and silent socialization. By contrast, the latter three approaches promote antiracism and resist dominant ideologies through the preparation for and validation of multiracial youths’ experiences with racial discrimination as well as explicit engagement with racism and antiracism in other ways (Csizmadia & Atkin, 2022).

Our findings build on previous theorizing and research (e.g., Atkin & Jackson, 2021; Bañales & Rivas-Drake, 2022; Csizmadia & Atkin, 2022; Hazelbaker et al., 2022; Lee et al., 2022; Miville et al., 2005; Root, 1990) in several ways to provide insights into how monoracial parents engage in myriad socialization strategies with their multiracial children. First, aligned with previous literature, parents’ own experiences with racism and their own understanding of racial injustice (Atkin & Yoo, 2019; Lorenzo-Blanco et al., 2013; Miller, 2020; O’donoghue, 2005; Rondilla et al., 2017; Talbot, 2008) informed their comfort level and capacity for addressing racism at the individual/interpersonal vis-a-vis the systemic level with their multiracial children. In addition, the child’s phenotype (Miville et al., 2005; Root, 1990; Talbot, 2008; Thekkedam, 2013), readiness for discussions about racism (Bañales & Rivas-Drake, 2022; Hazelbaker et al., 2022; Lee et al., 2022), and their own understanding of social injustice (Harris, 2016) played a role in monoracial parents’ engagement of antiracist socialization with their multiracial children.

The mothers in our sample had children with ages ranging from 2 to 25 years old. Although we did not explicitly ask parents how the age of their children influenced their socialization practices, it was apparent that for some, age does matter. Consistent with the work of Hughes (2003), several parents including Althea, Elena, and Tala engaged their children in more explicit conversations about racism and discrimination once their children entered adolescence, had themselves experienced racism, and/or began asking questions. However, Julia exemplified how parents can remain thoughtful about scaffolding antiracist conversations from a very young age as she sought out age-appropriate resources for her young children.

Moreover, we found that monoracial parents explicitly engaged a repertoire of strategies in their multiracial socialization. For instance, many relied on external sources of information about race and racism due to their lack of direct knowledge of what their children would experience growing up with multiracial rather than monoracial identities. This underscores the interconnectedness of familial multiracial socialization with that which happens outside the home. In the case of Jia and Chesa (approach 2), they acknowledged racism as existing but did not talk about this with their children and instead relied on other critical people in their children’s lives, such as teachers and peers (Miville et al., 2005), to provide them with information on race and racism. Similarly, we observed that parents who had thought about racism to a greater extent could and did draw on their own lived experiences with racism also relied on external sources of information to support their multiracial socialization efforts. Althea, Elena, Tala, and Julia variously referenced the use of media, books, and internet in addition to their own lived experiences, and they used these resources as a starting point to facilitate conversations with their children. This contrasts with Jia’s use of books as an *endpoint* for her child’s understanding of his father’s deportation. Jia offered her son these resources but then failed to discuss them further in the home, instead engaging with silent socialization.

Parents engaged in antiracist socialization in this sample also described a combination of self-reflection, modeling, conversations with partners or others, and engagement with supportive materials (e.g., books, media) to think about how racism may affect the lives of their multiracial children and other people. Several parents also viewed fostering the development of a positive multiracial identity as a way to resist negative societal or local community messages about their child’s self-worth. The blending of different multiracial ERS strategies among monoracial parents–most of whom were from racially minoritized groups–underscores the importance of having a repertoire of socialization practices to support the antiracism capacities of multiracial young people.

### Limitations and Future Directions

Although the current findings provide promising albeit preliminary evidence for five different approaches to socializing multiracial children towards an antiracist orientation, there are limitations that should be addressed through future research. One is that our sample of ten parents contained only mothers, and our protocol did not address how co-parents may have been involved in socializing their children towards antiracism. Future work would benefit from the perspective of fathers and other caregivers, as they may engage in qualitatively different multiracial socialization practices than mothers. Including all caregivers that reside in the household (e.g., mothers, fathers, grandparents) would further enrich our understanding as to what multiracial socialization messages children may be receiving from all critical people in the home (Miville et al., 2005; Renn, 2003; Root, 1990, 1996). This knowledge would help contextualize antiracist socialization efforts in multiracial households, especially if there are conflicting co-parenting messages about the nature of racism and how to navigate a racist society as a multiracial person.

Similarly, future work should address how other messages from critical peers, teachers, coaches, and the like may inform the development of an antiracist orientation in multiracial youth (Miville et al., 2005; Renn, 2003). As children develop in other critical places such as schools and neighborhoods, it is important that research address how socialization messages from sources external to the home may be impacting their beliefs about race and racism (Lorenzo-Blanco et al., 2013; Miville et al., 2005; Renn, 2003; Root, 1990, 1996). Multiracial youth learn about racism from a variety of sources, including peers and friends. As observed in this study, Ava and Chesa’s children were actively engaged in social justice movements even though this engagement was not socialized at home. The nature and ways in which youths’ experiences beyond the family may shape the nature of conversations and interactions around racism at home should thus be explored in future studies on antiracist multiracial socialization.

As noted, the larger SPARX study from which these data are drawn centered interaction between parents and their school-aged children. As a result, parents were asked to limit their discussion of their adult and/or infant children. This may have been a missed opportunity in the context of this research study. Parents may have explained how their practices differed for older (adult) or younger children if the protocol had not constrained their responses. Although the socialization practices outlined in our description of Julia’s practices show that antiracist socialization can begin when children are very young, it is not clear from our data how parents may adapt these practices as their children grow older. Future work can explore how parents may have adapted their approaches to antiracist socialization across their child’s lifespan and how approaches may have differed for siblings in the same household. Finally, this sample of parents is ethnically and racially diverse, offering insight into how these antiracist socialization approaches may occur across various multiracial households. However, we cannot reliably articulate how these socialization approaches occur within specific interracial contexts. For example, because there was only one Latino/Black household represented, we cannot assess how these socialization practices appear generally in multiracial Latino/Black households. Future research should assess how these socialization approaches occur within the confines of specific interracial households.

## Conclusion

A primary goal of the present study was to highlight ways monoracial parents could support their multiracial children’s capacities for antiracism. As recent years have sparked dialog regarding race, racism, immigration, and injustice, it is necessary that we draw from these findings to better understand how parents can raise “antiracist disruptors” who will go on to make changes to racist structures and systems. By better understanding how monoracial parents socialize race and racism more broadly to their multiracial children, we may be able to better shape the next generation of multiracial leaders to be advocates for social justice that are equipped to recognize and respond to racism and challenge dominant racist ideologies.

## Footnotes

1 This represents an increase of 24.8 million people from 9 million recorded in 2010.

2 Many of the RES practices discussed here have been well established as occurring in monoracial households prior to their application to multiracial families (e.g., cultural socialization, preparation for bias, egalitarianism; Hughes et al., 2006).

3 All names presented in this manuscript are pseudonyms and do not reflect the identities of any of the participants included in our sample.
